# Multisystemic resilience and its impact on youth mental health: reflections on co-designing a multi-disciplinary, participatory study

**DOI:** 10.3389/frcha.2025.1489950

**Published:** 2025-03-18

**Authors:** Linda Theron, Matteo Bergamini, Cassey Chambers, Karmel Choi, Olufunmilayo I. Fawole, Fyneface Dumnamene Fyneface, Jan Höltge, Thandi Kapwata, Diane T. Levine, Zainab Mai Bornu, Makananelo Makape, Celeste Matross, Brian McGrath, Olanrewaju Olaniyan, Dov J. Stekel, Josh Vande Hey, Caradee Y. Wright, Ameh Abba Zion, Michael Ungar

**Affiliations:** ^1^ Department of Educational Psychology, University of Pretoria, Pretoria, South Africa; ^2^ Shout Out UK, London, United Kingdom; ^3^ South African Depression and Anxiety Group, Johannesburg, South Africa; ^4^ Department of Psychiatry, Harvard Medical School, Harvard University, Cambridge, MA, United States; ^5^ Department of Epidemiology and Medical Statistics, Faculty of Public Health, University of Ibadan, Ibadan, Nigeria; ^6^ Youth and Environmental Advocacy Centre, Port Harcourt, Nigeria; ^7^ Resilience Research Centre, Dalhousie University, Halifax, NS, Canada; ^8^ Climate Change and Health Research Programme, Environment and Health Research Group, South African Medical Research Council, Pretoria, South Africa; ^9^ School of Criminology, Sociology and Social Policy, University of Leicester, Leicester, United Kingdom; ^10^ Centre for Social Development in Africa, University of Johannesburg, Johannesburg, South Africa; ^11^ School of History, Politics and International Relations, University of Leicester, Leicester, United Kingdom; ^12^ Regional Psychosocial Support Initiative, Johannesburg, South Africa; ^13^ Parsons School of Design, The New School, New York, NY, United States; ^14^ Health Policy Training and Research Programme, University of Ibadan, Ibadan, Nigeria; ^15^ School of Biosciences, University of Nottingham, Sutton Bonington Campus, Loughborough, United Kingdom; ^16^ Department of Mathematics and Applied Mathematics, University of Johannesburg, Auckland Park Kingsway Campus, Johannesburg, South Africa; ^17^ School of Physics & Astronomy, Centre for Environmental Health and Sustainability, University of Leicester, Leicester, United Kingdom; ^18^ Department of Geography, Geoinformatics and Meteorology, University of Pretoria, Pretoria, South Africa; ^19^ The Mandate Health Empowerment Initiative, Abuja, Nigeria

**Keywords:** African youth, mixed methods, multisystemic resilience, participatory design, youth depression

## Abstract

Youth depression is a global emergency. Redressing this emergency requires a sophisticated understanding of the multisystemic risks and biopsychosocial, economic, and environmental resources associated with young people's experiences of no/limited versus severe depression. Too often, however, personal risks and a focus on individual-level protective resources dominate accounts of young people's trajectories towards depression. Further, studies of depression in high-income countries (i.e., “western”) typically inform these accounts. This article corrects these oversights. It reports on the methodology of the Wellcome-funded R-NEET study: a multidisciplinary, multisystemic, mixed method longitudinal study of resilience among African youth whose status as “not in education, employment or training” (NEET) makes them disproportionately vulnerable to depression. Co-designed by academics, community-based service providers and youth in South Africa and Nigeria, with partnerships in the United Kingdom, Canada and the United States, the R-NEET study is identifying the physiological, psychological, social, economic, institutional, and environmental risks and resources associated with distinct trajectories of depression. Using the methodology of the R-NEET study as exemplar, this article advances an argument for understanding resilience as a contextually and culturally rooted capacity that draws on the multiple, co-occurring systems that young people depend upon to support their wellbeing. Acknowledging and harnessing the multiple systems implicated in resilience is critical to researchers and mental health providers who seek to support young people to thrive, and to young people themselves when protecting or promoting their mental wellbeing.

## Introduction

Despite recognition of the insidious and widespread impacts of depression on human wellbeing and socioeconomic development, attempts to prevent and manage this mental health crisis have been mostly inadequate (1). Addressing this crisis is especially difficult in low-and-middle-income countries (LMIC) where mental health is under-researched, mental health supports are scarce, help-seeking is stigmatised, and traditional cultural values (e.g., supportive interdependence) are waning (2, 3). Sub-Saharan African youth are disproportionately vulnerable: they live on a continent exposed to pronounced climate change threats and are predicted to become the largest youth population globally (4, 5). This combination will exacerbate livelihood challenges that are already fuelling African youth risk for depression (6). Moreover, at least 20% of African youth are not in employment, education, or training [i.e., NEET; (7). Being NEET imposes developmental “waithood” (8), p. 28], socioeconomic precarity, and psychological distress (9–11).

Given these threats, investigations of what might animate and sustain African youth resilience must be prioritised (6). It is crucial to do so in ways that do not reify trite person-focused accounts of African young people's capacity to sustain or regain mental health despite exposure to chronic stress (12). Instead, a more contextualized, multisystemic explanation of resilience is required that can inform the social, political, and economic solutions required to prevent depression among African youth. This goal informs the design of the Resilient-NEETs (R-NEET) study, an interdisciplinary, multisystemic, mixed method longitudinal investigation of resilience to depression among African youth who are NEET. In this paper, we detail the R-NEET methodology and use that methodology to advance an argument for understanding resilience to depression as a contextually and culturally rooted capacity grounded in multiple, co-occurring systems.

## The multisystemic roots of resilience to depression

Shifting the focus from a single system when accounting for resilience aligns with the ongoing global attention to the multiple determinants of mental health (13), including related arguments advanced by the UN Rapporteur for the Right to Mental Health. These arguments foreground that mental health and wellbeing are rooted in a “social, psychosocial, political, economic and physical environment that enables individuals and populations to live a life of dignity, with full enjoyment of their rights and in the equitable pursuit of their potential” (14). Likewise, a narrative review of the factors contributing to mental ill-health among young people underscored that multiple determinants—especially social, economic and commercial ones—put youth at risk for poor mental health outcomes (15).

Young people who are exposed to the above risks but avoid poor mental health outcomes (e.g., report no or minimal symptoms of depression) are thought to show resilience (16). While the capacity to adapt well to significant stress exposure has traditionally been equated with psychological ruggedness, more recent explanations of resilience discourage simplistic, mono-systemic explanations of young people's ability to be mentally healthy despite exposure to substantial stress (17–22). Explained differently, youth resilience cannot be attributed to a single system or single aspect of a young person's life or personality. Instead, and as conceptualised in the R-NEET study, resilience is a responsive multisystemic process—i.e., one that is distributed across physiological, psychological, social, institutional, and environmental systems—that fits specific situational and cultural dynamics at a given point and over time.

Psychological strength remains important to resilience, but biological, social, structural, and environmental resources are understood to matter just as much (16, 17, 23–29). For example, a study with 290 children with experience of AIDS-related orphanhood showed that at least a quarter of these children had no symptoms of mental illness (30). Their resilience was attributed to personal attributes (i.e., physical health), as well as social and environmental resources (i.e., quality caregiving, food security, quality peer relationships, and living in a community that did not expose them to high levels of violence). Likewise, during the COVID-19 pandemic, the wellbeing of Australian young adults was bolstered not only by psychological, but also by social and environmental resources. This combination included hopefulness, secure employment, positive (virtual) social contact, and accessible outdoor spaces (e.g., a garden) (31).

In addition to illustrating the multiple systems that matter for mental health resilience (e.g., reporting no or limited symptoms of depression despite exposure to significant stress), the above examples focus attention on the contextual, developmental, and temporal dynamics that determine which resources matter most for mental health resilience. While a detailed understanding of the resources that support resilience to depression is crucial to the prevention and management of the disorder (32), it is equally important to identify what combination of these resources matters most for which group of young people in which context and what point in time (14). Like other studies of mental illness, depression-focused studies have typically not done this. These studies have not only paid too little attention to systemic resilience factors, but also neglected their time scale and objective measurement. For the most part, studies of depression have focused on heterogeneous risks (33). When resilience-enabling factors are considered, psychological strengths such as self-esteem, positive emotions, and self-regulation are often foregrounded (34–36).

Some depression studies acknowledge the resilience-enabling value of other systemic factors (37, 99). These include food security (38); physical health/activity (which are often reliant on access to health care or spaces and places to exercise (39–41); less polluted, more temperate environments and green spaces (42–44); trusted social connections (45, 46); and opportunities for social justice (47). Nevertheless, these studies typically do not clarify which combination of resilience-enabling factors will have a pronounced protective effect on which young people's mental health. They also do not clarify how culture and context shape our understanding of which factors matter more/less (37).

An exception to the emphasis on psychological ruggedness and inattention to resource combinations can be found in the Resilient Youth in Stressed Environments (RYSE) study (48). This study, which included several of the R-NEET investigators, examined what supports the mental health resilience of 1,072 14-to-24-year-olds living in oil and gas dependent (i.e., economically volatile, migrant) communities in Canada and South Africa. It used multisystemic and network modelling approaches to discover resource combinations that scaffold positive mental health outcomes (22, 49, 50). Specific to depression outcomes, RYSE reported protective combinations of (i) cultural and spiritual resources for Canadian and South African (SA) youth (51); (ii) caregiver warmth and monitoring for SA youth (52); (iii) individual and social resources for Canadian youth (53); and (iv) physical, social, institutional and ecological resources for SA youth with elevated vs. low depression trajectories (19). While this work is promising, it lacked repeated, objective measurement of protective physical health and environmental resources (e.g., indoor temperature, indoor air quality; accessible green space). It also lacked sufficient time points to predict combinations of protective factors or gauge seasonal (i.e., temperature) effects. As explained next, the design of the R-NEET study addresses these limitations and advances attention to contextually responsive combinations of resilience-enabling factors that have a pronounced protective effect on young people's mental health at multiple time points.

## The R-NEET study methodology

Funded by Wellcome Trust, the R-NEET study is a 66-month investigation that commenced on 1 February 2024. Framed by multisystemic understandings of resilience (17, 18, 21, 27, 29) and co-designed by academics, community-based service providers and youth in SA and Nigeria, with partnerships in the United Kingdom, Canada and the United States, the R-NEET study is identifying the constellation of physiological, psychological, social, economic, institutional, and environmental risks and resources associated with distinct trajectories of depression among young people who are NEET. To that end, the study seeks to answer two overarching questions: (i) What networks of multisystemic factors enable/constrain resilience to depression at multiple timepoints for sub-Saharan African youth who are NEET and living in stressed communities (i.e., low income, polluted, and/or exposed to violence)?, and (ii) How do these networks differ by a young person's social and physical positioning (e.g., sociodemographic status including gender and sex, geographical location, mobility/access to transport, migration status, etc.)?

**Figure 1:**
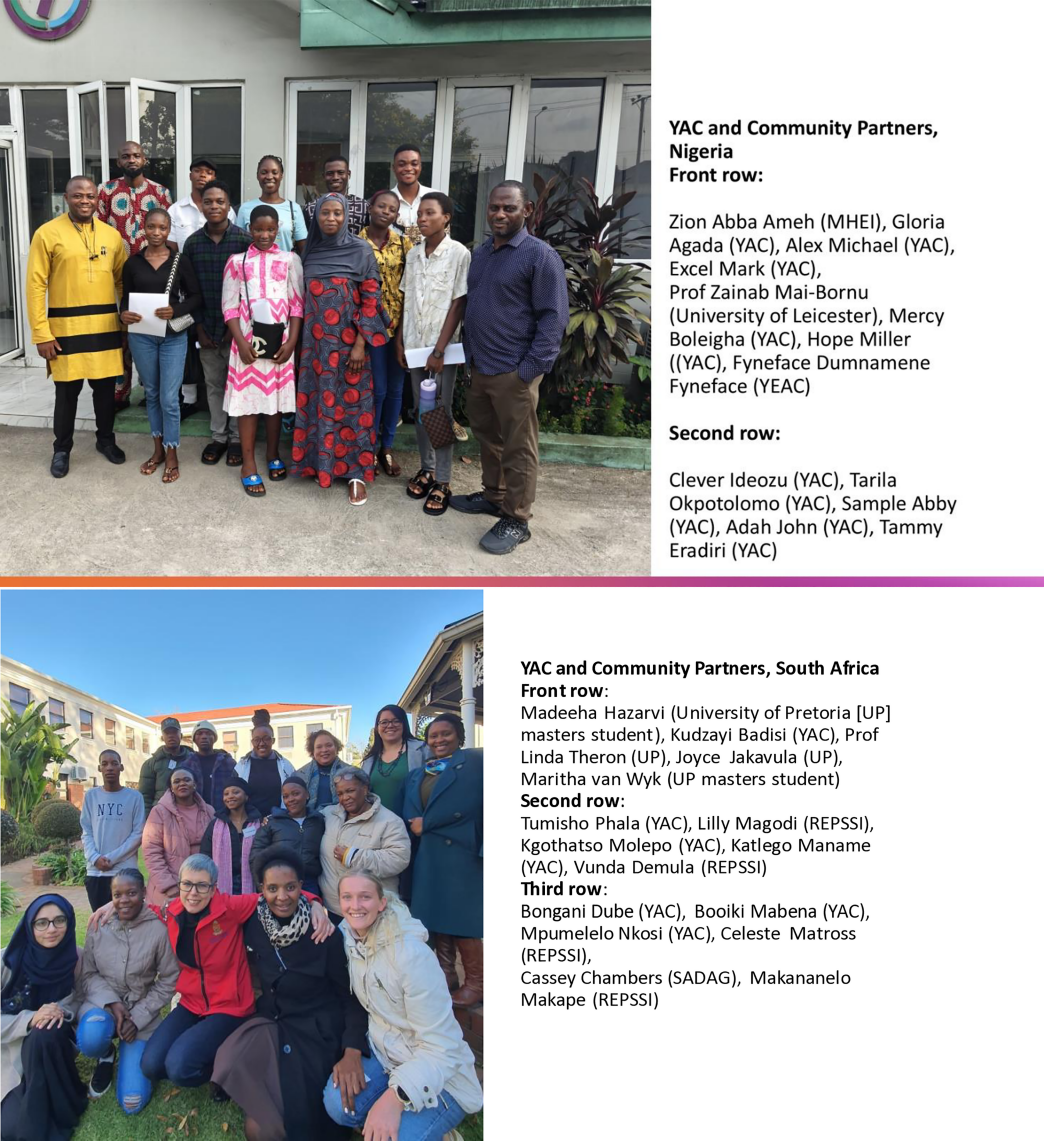
YAC and community partners, Nigeria & SA.

Drawing on our experience in the RYSE study (48), co-design is fundamental to advancing study uptake and mobilisation of the answers to the above questions (54–56). Given R-NEET's focus on depression among youth who are NEET, we sought partnerships with stakeholders who are renowned for their long-standing commitment to youth mental health and who maintain extensive networks of youth, service providers, and policy makers. These partners—i.e., Mandate Health Empowerment Initiative (MHEI), Nigeria; Regional Psychosocial Support Initiative (REPSSI), SA; the South African Depression and Anxiety Group (SADAG), SA; and Youths and Environmental Advocacy Centre (YEAC), Nigeria—co-developed the purpose and methodology of the R-NEET study. They also invited local youth to contribute to the study's co-creation (57, 58), before establishing Youth Advisory Committees (YAC; Figure 1). YAC eligibility was dependent on five criteria: age (18–24 years); experience (past or present) of NEET-hood; English literacy; residence in an R-NEET study site; and track record of commitment to supporting the wellbeing of local youth and positive youth leadership qualities. As in other youth-focused studies (48, 59), YACs are integral to the operationalisation of the R-NEET study, including advancing ethical sensitivity, supporting participant recruitment, refining study instruments, enabling rich contextualised understandings of the results, and mobilising knowledge.

### Context

The R-NEET study is located in Nigeria and SA as both these countries report elevated NEET rates, with 43.2% of 15- to 35-year-olds identifying as NEET in SA (60) and 36.6% in Nigeria (61). Further, both countries are characterised by high levels of inequality and corruption (62), limited mental health services (63, 64), and increased exposure to climate change-related environmental risks and high levels of air pollution (65), all factors at different systemic levels believed to contribute to depression (33).

In SA, the setting is low-income, violence-exposed communities (e.g., townships) in any of the three major municipalities in Gauteng Province: City of Ekurhuleni, City of Johannesburg, and City of Tshwane. Many youths migrate to Gauteng (SA's wealthiest province), but there are concerns about their wellbeing given their “general sense of hopelessness” (66). This hopelessness is fuelled by rampant unemployment, poor service delivery and related violent protests, high crime rates, exposure to environmental health risks, and declining quality of life (67, 68).

In Nigeria, the location is low-income, violence-exposed communities in Rivers and Bayelsa states in the Niger Delta in the South-South geopolitical zone. Despite vast wealth associated with oil fields, youth in the Niger Delta report distress relating to economic exclusion, environmental degradation, and conflict (69, 70).

Both sites are vulnerable to climate emergency impacts, with emphasis on thermal threats. In addition to the general warming that is being experienced across the globe (71), there are concerns about the impact of growing urban populations on the surface and air temperatures in sub-Saharan Africa (including the R-NEET sites in Gauteng and the Niger Delta; (72). Rising temperatures jeopardise physical and mental health, as well as livelihoods and food security (71).

### Sample

The R-NEET study will recruit 1,600 participants. To that end, the study will be advertised via flyers displayed in places that are popular with youth (e.g., taxi ranks, spaces where youth exercise, faith-based organisations). The flyers will also be distributed electronically via partners' extensive social media networks and listservs and communicated verbally at community events (e.g., mental health workshops; stakeholder meetings).

Using a purposive sampling approach, study participation will be invited from 18- to 24-year-olds who are NEET (for at least 2 weeks prior to first participation in the study), have basic English literacy and communication skills, and live in a R-NEET study site. The stipulated period of NEET-hood (i.e., 2 weeks prior to first participation) relates to the fluidity of NEET status (11); we anticipate that this status will fluctuate over the course of the study. Youth who are homeless, or whose capacity to consent is impaired, will be excluded. Studying the experiences of youth who are NEET focuses our work on one especially vulnerable population that is common across Africa and diaspora communities (9).

### Research plan

**Figure 2:**
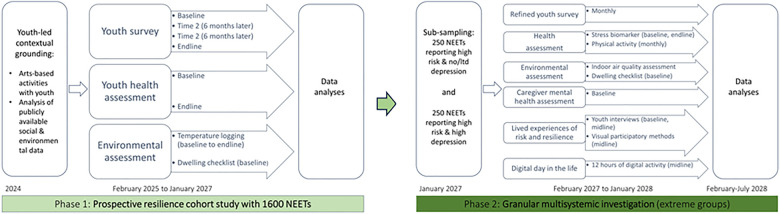
Summary of R-NEET research phases and activities.

The R-NEET study comprises two distinct research phases, each comprising concurrent data collection and analyses (see Figure 2).

#### Phase1: prospective resilience cohort study

Phase 1 runs from February 2024 to January 2027 and involves the entire cohort (*N* = 1,600 NEETs). The principal investigator's internal review board (IRB, i.e., the Faculty of Health Sciences Research Ethics Committee and Faculty of Education Ethics Committee, University of Pretoria) has provided ethical clearance for Phase 1 (EDU 166/23); co-investigator IRBs are providing aligned clearance. In keeping with the multisystemic resilience framework of the R-NEET study (17, 18, 21, 27, 29), and the heterogeneous risk and resilience factors associated with depression (1), Phase 1 will identify the physiological, psychological, social, economic, institutional, and environmental risks and resources that play into participants' vulnerability or resilience to depression at a point in time and over time (i.e., both cross-sectionally and longitudinally).

##### Youth-led contextual grounding

**Figure 3:**
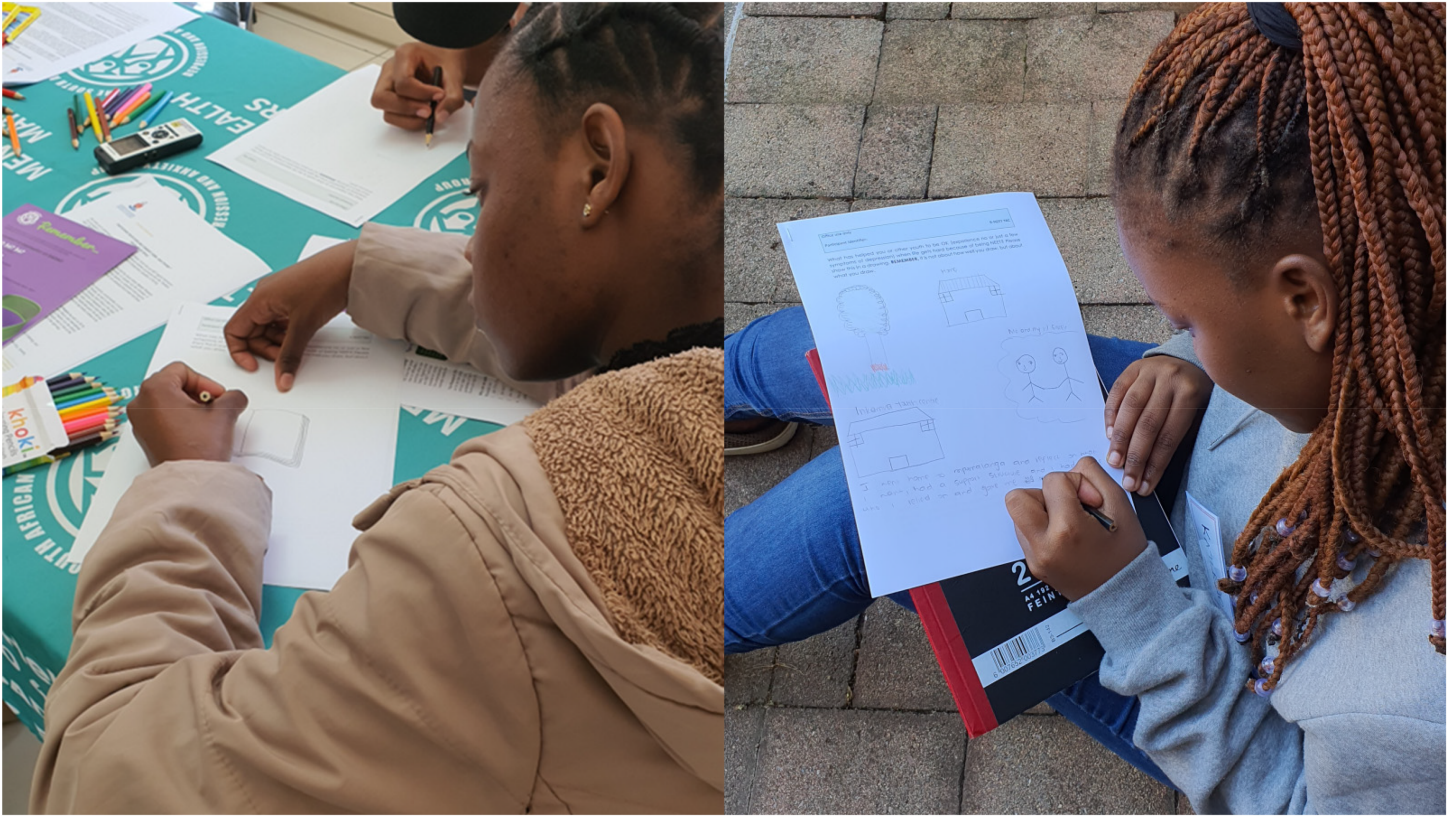
YAC engaging in Draw & Write research activities.

Using arts-based activities (e.g., draw-write-talk; see Figure 3) and YAC-led transect walks (i.e., a walk that reveals the location of risks and resources; (48), YACs are in the process of contextualising the study by sharing their lived experience of local factors that enable/constrain youth resilience to depression. As is typical in participatory work (73), the visual artefacts and walks are being used to elicit conversations with young people to co-develop rich nuanced understanding of how local context plays into risk and resilience.

The youth-led contextualisation will be supplemented with analyses of publicly available social and ecological (i.e., spatial-temporal ground-based, satellite, and GIS) data. We are investigating indoor temperature; ambient (outdoor) temperature and air quality; blue/green/recreational space availability; transportation networks; service-provision; crime statistics; NEET statistics; and local social media trends.

Using a realist evaluation approach (what works for whom in which context and why; (74), and as pre-registered in the International Prospective Register of Systematic Reviews (PROSPERO) database (registration number: CRD42023440153), we are reviewing peer-reviewed publications documenting physiological, psychological, social, institutional, environmental, and cultural resources associated with lower levels of depression. This review will shed light on the depth and breadth of understanding of the multisystemic factors that matter for resilience to depression, along with contextual relevance of this evidence base for supporting resilience to depression among African youth who are NEET or otherwise socioeconomically vulnerable.

Taken together, the above will facilitate co-created risk/resilience assumptions that will inform later network analyses of data drawn from the multiple co-occurring systems implicated in youth resilience.

##### Youth surveys

Building on the understanding that resilience studies must investigate risks, resources *and* an outcome of researcher choice (14), the survey will assess self-reported (1) risk factors (e.g., NEET status, personal lifestyle risks; social media use; adverse family events; community violence; food insecurity; political distrust); (2) resilience-enablers [e.g., psychological strengths, social (family, peer, community) supports, cultural heritage, institutional supports, access to enabling financial support]; and (3) a mental health outcome (depression symptoms, as measured by the Patient Health Questionnaire [PHQ-9; (75); the PHQ-9 has been shown to be a valid measure in both South African and Nigerian depression studies, (76).

The survey, which totals 182 items, draws on the RYSE self-report measure of youth risk and resilience (a compendium of validated scales and sub-scales, (48) and the above-mentioned systematic review. It was modified following YAC consultation (e.g., the YAC directed the inclusion of contextually relevant questions, such as reliance on “loan sharks” [informal money lenders that charge high interest) and cognitive interviewing (i.e., NEET youth described their interpretations of questions, offer suggestions for improvement). The revised survey battery was piloted (30 NEETs/country). In addition to a section measuring the outcome of interest (i.e., depression, measured with the PHQ9; (75), the finalised version includes three sections. These are: demographic details; risk exposure [including 4 validated scales/sub-scales/and self-developed items about family adversity, neighbourhood stressors and (lack of) social cohesion, social comparison, perceived stress and NEET-related stressful events, and financial risks]; and resilience-enabling resources (including 6 validated scales/sub-scales and self-developed items about personal, relational/social and contextual resilience supports, positive emotion, religious beliefs, self-compassion and social media use). The survey will be administered 4 times to the R-NEET cohort [see Figure 2 for time points, starting in February 2025 (baseline) and spaced 6 months apart].

##### Youth health assessment

Youth will complete two physical health assessments. Health professionals will measure heart rate, blood pressure, body mass index, and eyesight at baseline (February-July 2025) and endline (August 2026-January 2027). They will also administer a brief health questionnaire about health-related habits, heat-related illness, and health-related quality of life. The repeat assessments will document young people's health over time (i.e., sustained/improved/worsening health).

##### Environmental assessment

Using temperature loggers (remote data logging tools), we will assess thermal comfort by measuring indoor temperature (and relative humidity to calculate Apparent Temperature, or “real-feel” temperature associated with heat-related health outcomes). This will be done from baseline to endline of Phase 1. We will also document environmental health determinants at baseline. This includes dwelling characteristics (type of dwelling and building material/roof/windows/floor/ceiling; shade trees near dwelling; number of rooms; cooking/heating/lighting fuel options, including energy alternatives during power outages; water/sanitation; presence of damp/mould; presence of environmental tobacco smoke/pets; measures for cooling; and ventilation), number of people sharing the dwelling, and access to water and sanitation.

##### Analysis of phase 1 data

We will use the data collected in Phase 1 to develop and analyse a multisystemic network predictive of depression trajectory outcomes, calibrate the network on our data, and use that network to identify those factors to which depressive outcomes are most sensitive. We plan to use probabilistic graph models because they can describe a multilayer, multisystemic influential structure, and will enable us to use methods from causal inference/Bayesian networks including network structure inference and counterfactual analyses. Specifically, we will: (1) Define dynamic risk and resilience factor profiles by using parallel-process growth mixture modelling to identify multisystemic dynamic trajectory profiles of the risk/resilience factor measures from the four survey iterations. (2) Define the outcome by identifying distinct trajectories of depressive symptoms, also using growth mixture modelling with the survey data. Based on RYSE results (20) and prior landmark studies (77, 78), we expect to identify at least four depression groups (stable low; declining; worsening; chronic high). (3) Define a multisystemic network model designed to explain the depressive trajectory outcomes, that will integrate multisystemic data from the dynamic risk and resilience factor profiles (surveys), the physical health profiles (health assessment), the physical dwelling profiles (environmental assessment), and the processed indoor environment data (environmental assessment). Two approaches will be taken to determine network structure: (i) building the network structure using co-created outputs of risk/resilience determinants (informed by activities in the youth-led contextual grounding); and (ii) performing network inference on the data using suitable statistical packages for causal inference/Bayesian networks. Network parameters (i.e., conditional probabilities) will also be inferred using these packages. (4) The network model itself will be built in a compositional way. Each node in the network represents a (relevant) measured variable (or composite e.g., from a scale): the values that a node takes are described by a probability distribution with parameters that depend on the other nodes in the network that feed into it. Those distributions will be based upon multi-level general linear model regression (to account for both continuous or categorical factors as appropriate, and nested data) between factors as determined by the network structure. For example, we will employ moderated network models and meta-analytic Gaussian graphical models to investigate the influence of community- and country-level variables on individual-level networks (79, 80). (5) Identify high sensitivity factors using a parameterized network model that will be subject to analyses to identify those in the network to which depressive outcomes are most sensitive. A wide range of other available methods will be evaluated including heterogeneous treatment effect estimation, counterfactual analyses, and decision tree methodology. For example, we will also explore latent profile analysis as well as other unsupervised machine learning/AI techniques on a wider range of measures to identify groups of participants with more broadly similar profiles and examine how these data-driven profiles relate to depression which will be useful in development of interventions and participant-facing materials.

#### Phase 2: A granular multisystemic investigation with extreme groups

Phase 2 runs from February 2027 until July 2029. From youth reporting chronic high-risk exposure in Phase 1, we will sample two extreme sub-groups: those reporting high risk and no or minimal symptoms of depression (*n* = 250) vs. elevated symptoms of depression (*n* = 250). These extreme groups will be invited to complete Phase 2 empirical work (February 2027-January 2028; see Figure 2).

##### Refined youth surveys

The sub-sampled extreme groups will complete monthly surveys (x 12). The Phase 1 survey will be reduced to the most influential risk and resilience factors as identified in the youth-led contextualisation (and related qualitative work) and preliminary Phase 1 data analyses. The refined survey will retain the PHQ-9 to measure symptoms of depression.

##### Air quality assessment

We will repeat Phase 1 measures for environmental factors that were most influential. Given the association between air quality and youth depression (43), we will also assess indoor-dwelling air quality using low-cost sensors that measure particulate matter (PM2.5 and PM10) at 10 min intervals.

##### Stress biomarkers and activity levels

As in RYSE (48), we will assay non-invasive biomarkers (hair cortisol and DHEA levels) and identify an integrated index of cumulative cortisol exposure over time. We will collect two hair samples (baseline [February 2027] and endline [January 2028). Most SA RYSE participants (majority Black African) provided hair samples once we had explained the rationale and process to them in detail (48); lessons learned during RYSE will advance how the R-NEET team will collect and assay hair samples.

Using wearables, we will track participant activity levels. While high levels of physical activity are associated with lower risk of depression (45), it is unclear if this association will hold for African youth in resource-constrained communities where physical activity (e.g., walking to public transport hubs) is a default routine. We will also ask youth to self-report physical activity in order to differentiate recreational and commute-related physical activity.

##### Assessing caregiver mental health

RYSE taught us the importance of caregivers for older youths' resilience to depression (51). Caregiver wellbeing impacts caregiving quality (81). To better understand caregiver contributions to resilience among emerging adults who are NEET, and in keeping with African embracing of flexible kinship (82), we will invite participants to self-identify a primary caregiver. Following consent, we will administer the Self-Report Questionnaire (SRQ-20) to better understand caregiver mental health/stress exposure.

##### Exploring lived experiences of risk and resilience

Using a Reflexive Thematic Approach (83), we will interview youth to better understand their lived risk/resilience experiences. YACs will co-develop the individual semi-structured interview protocols that will probe risks and resources across multiple systems. While theoretical saturation will determine sample size, we anticipate 20 youth per extreme group/country (*N* = 80). To better understand risks and resources over time, the same participants will be interviewed six months after the first interview.

We will also invite five youth per extreme group/country to help us better understand their lived risk/resilience experiences by engaging in arts-based or digitally-mediated methods that are valuable for exploring young people's context-responsive interactions with their social and physical ecologies and for representing these in culturally responsible, decolonised ways. In our previous studies this tool basket included: Draw-and-Write (84); video recording of a “day-in-the-life” (85); digital storytelling and/or digital diaries (86, 87); and participatory video (88, 89).

##### A digital day-in-the-life

Working with 10 young people per extreme group, we will document 12 h of participants' technology use. To that end, we will shift the focus of the original “day-in-the-life” away from routine in-person activity (85), to routine virtual activity. This digital day-in-the-life will advance our understanding of young people's daily routines, behaviours and practices within digitally-mediated environments. Screen-recorded data will then be correlated with environmental and biological data gathered during the same time period, and social media/crime trends for the week in question. Environmental data will be drawn from Earth observation and GIS data for the day in question (specifically land use/cover in the area, weather, air quality, and transportation networks), and biological data from wearable technologies work during the DDitL. A follow-up clarifying interview will be held with each participant. As well as feeding the data into the network model, we will use aggregated data during focus groups to create compilations of the themes emerging, and share these across sites to enable cross-cultural commentary.

##### Analysis of phase 2 data

We will produce and analyse refined and extended multisystemic network graph models for depression using data from Phase 2 in order to account for differentially impactful resource combinations that protect NEET youth from reporting elevated levels of depression at higher levels of chronic risk. This will involve three approaches: (1) repeating the Phase 1 analysis methodologies to include the additional data; (2) applying causal time series analysis on the detailed longitudinal data from Phase 2, making use of linear dynamical systems using both classical statistics (e.g., Grainger Causality) and Bayesian approaches (i.e., Bayesian Structural Time Series Models); (3) following a concurrent nested approach (90), we will analyse the qualitative data from Phase 1 and Phase 2 separately and nest the identified themes in the quantitative results of Phase 2 as a lens through which to interpret the identified themes. All of these steps to our analysis plan will ensure a synthesised and contextualised set of findings.

### Expected outcomes

Phases 1 and 2 described above will blaze a methodological trail that is likely to develop significant new understanding of the combined role of physical/biological, psychological, social, institutional and environmental factors associated with African youth resilience to depression over time. In doing so, we will transform the traditionally narrow focus on personal sources of youth resilience (also to depression), and the under-investigation of youth mental health in LMICs. Applying a multidisciplinary approach to discovering which resilience mechanisms matter most for which youth in which context will identify multisystemic pathways that will further precision medicine and advance social interventions to prevent and manage depression. Uniquely, this work will redress the dominance of high-income country (HIC) or “western” accounts of youth resilience and be the first to examine concurrently the multiple systemic factors associated with the resilience of African youth exclusively.

## The case for a contextually responsive multisystemic approach to depression research and management

The R-NEET study, which is grounded in co-created and participatory mixed methods, has the potential to provide a blueprint to guide thinking on how best to conceptualise studies of depression that will yield more detailed and comprehensive insights on multisystemic drivers of resilience in at-risk young people. Bonanno (78) is unequivocal that “any attempt to build or enhance resilience that focuses on one or even a few of the known correlates will likely be inefficacious” (p. 2). Concurrently and repeatedly measuring a wide range of physiological, psychological, social, economic, institutional, and environmental risks and resources associated with distinct trajectories of depression among young people is a first step to redressing Bonanno's concerns. Moreover, the R-NEET study's emphasis on discovering what combination of the above-mentioned factors matters most for which groups of young people at a point in time and over time redresses the longstanding foregrounding of HIC studies of resilience that typically emphasise personal strengths. Not only is this emphasis at odds with traditional African valuing of the collective rather than the self (91), it also advances dissatisfaction with resilience understandings that place the onus on the individual (rather than the system) to facilitate change (12, 92).

A study of multisystemic factors associated with resilience to depression, however, is complicated not only by the over-emphasis on individual protective factors in the current scientific discourse, but also by the complexity of accounting for multiple systems in the same analysis. Measuring psychological health and indoor temperature (as we do in R-NEET) may make theoretical and practical sense based on previous investigations of their association, but standardizing data and identifying appropriate ways to analyse those data raises ontological questions and poses statistical problems. Can we be confident that factors associated with resilience at different systemic levels are actually related, and how does one conduct network analyses to demonstrate factor co-dependency? From a qualitative point of view, are the data trustworthy; specifically, do the data have enough face validity to justify investigation or is analysis of co-occurring systems nothing better than a fishing expedition in hopes of finding a random association? Our previous work with multisystem data suggests that solutions are possible (22). R-NEET, though, corrects many of the shortcomings of our own earlier studies, introducing more time points for a small subsample of the population, enhancing environmental tracking, and growing the sample size to the point where we can achieve greater predictability for outcomes like depression. Better predictability of depression trajectories and the risk and protective factors associated with them is key to prevention and optimal management of youth depression and its associated suffering.

It is also worth noting that a study of this scope is occurring in two LMICs (SA and Nigeria) rather than an HIC (i.e., Canada or the UK), allowing us to hopefully reverse the pattern of influence so common in the psychological sciences: typically, it is HIC-generated concepts and methods that inform research in LMIC. R-NEET is reversing this flow of knowledge, removing the trend to colonise research in the Global South by creating the basis for future studies of resilience in the Global North that rely on the conceptual and methodological advances of a study like R-NEET. Specifically, R-NEET challenges the epistemological and ontological traditions common in HIC which have decontextualized our understanding of positive developmental processes (93, 94).

While we are confident that the contextually responsive multisystemic approach to depression research and management outlined in this article is an important way forward, we remain mindful that a single adaptive outcome (e.g., no or low symptoms of depression in the face of NEET-related challenges) does not necessarily constitute resilience (95). In contexts, like Africa, where young people repeatedly navigate multiple challenges, their capacity to adapt could also be reflected in outcomes such as peacekeeping, civic engagement, or community building, or even a combination of these (96). Still, regardless of the outcome used to denote resilience, its realisation requires that researchers, practitioners, service providers, and other stakeholders have a profound understanding of the complex multidimensionality of the risks and resources underpinning it—the R-NEET multisystemic, iterative methodology does just that. We are hopeful that our use of this methodology, albeit with a single mental health outcome, will inspire studies of the multisystemic pathways that inform other adaptive outcomes or a combination of outcomes. In so doing, it is likely that the traditional and inadequate preoccupation with the psychosocial factors implicated in young people's outcomes will be helpfully expanded (97), including recognition of social, economic and commercial factors (15), as well as environmental ones (98).

## Conclusion

Depression has been characterised as “avoidable suffering” (1), p. 124). But, avoiding depression hinges on researchers identifying the constellation of risks and resources—within and across systems—that matter for mental health. Though achieving a better understanding of youth resilience to depression is not (yet) certain, our co-designed R-NEET methodology is already helping to clarify the challenges of conducting multisystemic research on protective processes. It promises a contextualized, multisystemic explanation of resilience that can inform the social, political and economic solutions required to prevent depression among African youth and catalyse similarly multipronged solutions elsewhere.

## Data Availability

The article reports a novel methodology, it reports no data. The data that this methodology will generate are not reported in this article. When the R-NEET study is complete, the data will be made publicly available via Figshare.
